# Application of Amniotic Membrane in Skin Regeneration

**DOI:** 10.3390/pharmaceutics15030748

**Published:** 2023-02-23

**Authors:** Nurul Fitriani, Gofarana Wilar, Angga Cipta Narsa, Ahmed F. A. Mohammed, Nasrul Wathoni

**Affiliations:** 1 Department of Pharmaceutics and Pharmaceutical Technology, Faculty of Pharmacy, Universitas Padjadjaran, Jatinangor 45363, Indonesia; 2 Pharmaceutical Research and Development Laboratory of FARMAKA TROPIS, Faculty of Pharmacy, Universitas Mulawarman, Samarinda 75119, Indonesia; 3 Department of Pharmacology and Clinical Pharmacy, Faculty of Pharmacy, Universitas Padjadjaran, Jatinangor 45363, Indonesia; 4 Department of Pharmaceutics, Faculty of Pharmacy, Minia University, Minia 61519, Egypt

**Keywords:** amniotic membrane, skin application, stem cells

## Abstract

Amniotic membrane (AM) is an avascular structure composed of three different layers, which contain collagen, extracellular matrix, and biologically active cells (stem cells). Collagen, a naturally occurring matrix polymer, provides the structural matrix/strength of the amniotic membrane. Tissue remodeling is regulated by growth factors, cytokines, chemokines, and other regulatory molecules produced by endogenous cells within AM. Therefore, AM is considered an attractive skin-regenerating agent. This review discusses the application of AM in skin regeneration, including its preparation for application to the skin and its mechanisms of therapeutic healing in the skin. This review involved collecting research articles that have been published in several databases, including Google Scholar, PubMed, Science Direct, and Scopus. The search was conducted by using the keywords ‘amniotic membrane skin’, ‘amniotic membrane wound healing’, ‘amniotic membrane burn’, ‘amniotic membrane urethral defects’, ‘amniotic membrane junctional epidermolysis bullosa’, and ‘amniotic membrane calciphylaxis’. Ultimately, 87 articles are discussed in this review. Overall, AM has various activities that help in the regeneration and repair of damaged skin.

## 1. Introduction

The skin is one of the largest organs in the body, and it performs various functions, including defense against pathogens, protecting against mechanical damage, regulating body temperature, and maintaining fluid balance [1,2]. Disruption of the structural integrity and normal function of the skin caused by trauma can cause wound formation [3]. Normal wound healing is a physiological response to tissue damage; this process involves four stages: hemostasis, inflammation, proliferation, and remodeling. During hemostasis, the blood clot controls blood flow and forms the primary matrix in which cells migrate. During the inflammatory phase, monocytes and lymphocytes are induced to transmigrate to the wound base; consequently, the secretion of cytokines and growth factors that activate fibroblasts is stimulated. During the proliferative phase, fibroblasts embedded in the thrombus proliferate and secrete transient extracellular matrix (ECM). The ECM contributes to the formation of granulation tissue and the germination of new blood vessels and maintains the viability of the new tissue. At the same time, keratinocytes lose contact with the basement membrane and are pushed to migrate toward the wound space. The process of reepithelialization involves the granulation tissue and the scab. Finally, during the remodeling phase, the wound contracts and forms a scar. This step includes ECM remodeling and apoptosis of fibroblasts and macrophages [4,5].

Skin regeneration refers to treatments that are more reconstructive and aimed at repairing complex tissues. They are more suitable for chronic injuries that do not benefit from other invasive strategies, such as increased application of dressings or follow-up treatments using physical/mechanical methods. Skin regeneration products represent the most innovative skin injury care sector and involve a multidisciplinary approach [6]. Various methods and wound dressings are available to enhance and speed up the wound healing process, including the use of an amniotic membrane (AM) [7]. The AM is the layer closest to the placenta; it has a unique molecular structure and is invulnerable to immune system reactions [8]. Its cellular components actively suppress immune cell activity through paracrine secretions that cause the amniotic membrane to exert an immunomodulatory effect on implantation and can prevent the rejection of its cellular charge [9]. The AM is one of the biomaterials showing great potential for use in tissue engineering, especially in the skin [8,9]. This review article aims to identify the application of AMs on skin regeneration.

## 2. Method

This review involved searching for published research articles in several academic databases, namely Google Scholar, PubMed, Science Direct, and Scopus. The papers were published between 2009 and 2022. The searches included the keywords ‘amniotic membrane skin’, ‘amniotic membrane wound healing,’ amniotic membrane burn,’ ‘amniotic membrane urethral defects,’ ‘amniotic membrane junctional epidermolysis bullosa’, and ‘amniotic membrane calciphylaxis.’

## 3. Structure and Composites of Amniotic Membrane

AM is the innermost layer of the placenta adjacent to the fetus. It consists of a single layer of epithelium, a thick basement membrane, an avascular layer, and a stromal matrix with a thickness of 0.02–0.5 mm [10,11,12]. The epithelial layer is a layer of metabolically active cuboidal and columnar cells that are in direct contact with the amniotic fluid [12,13]. The basement membrane consists of ECM components, including various types of collagen, elastin, laminin, fibronectin, and vitronectin, which are non-collagenous glycoproteins; glycosaminoglycans such as hyaluronic acid; natural inhibitors (matrix metalloproteinases [MMPs]; and biologically active factors, namely cytokines and growth factors that are beneficial for wound healing and tissue regeneration [14,15]. Several growth factors—basic fibroblast growth factor (b-FGF), epidermal growth factor (EGF), keratocyte growth factor (KGF), and hepatocyte growth factor (HGF)—are known to modulate the wound healing response [16]. The stromal matrix or mesenchymal layer is divided into three adjacent layers. There is a dense inner layer that is in contact with the basement membrane and contributes to the tensile strength of the membrane, a thick intermediate fibroblastic layer consisting of loose fibroblastic tissue, and a spongy outer layer. The AM outer layer consists of fibroblast-like mesenchymal cells. It probably originates from the embryonic mesoderm plate and is fully distributed in AM. The content of the collagen-rich mesenchymal layer increases its tensile strength [12,13,17].

## 4. Preparation Method of Amniotic Membrane

AM is obtained from human placenta collected by medical personnel from pre-selected donors following cesarean section after undergoing selective screening with negative serology for human immunodeficiency virus (HIV), hepatitis C virus (HCV), hepatitis B virus (HBV), cytomegalovirus, and syphilis, and negative molecular tests for HIV-1, HIV-2, HBV, HCV, and microbes [18,19,20,21]. For cryopreservation, immediately after delivery, the placenta is washed with 0.9% NaCl solution and transferred to a tissue bank at +4 °C. AM is separated and washed with 0.9% NaCl solution. After removing all clots adhering to AM, it is placed in an antibiotic solution (500 mg/L amikacin, amoxicillin 1 g/L, vancomycin 500 mg/L, and amphotericin B 50 mg/L) for 2 h at room temperature. Then, after washing with 0.9% NaCl solution, AM is cut into 9 cm^2^ pieces. Each fragment is placed on a nitrocellulose membrane and transferred to a petri dish containing RPMI 1640 media with 17% glycerol (as a cryoprotectant). Petri dishes are placed in sterile bags, sealed, and stored at −80 °C until use. One hour before application, cryopreserved AM is thawed at room temperature. After rinsing with a balanced saline solution (BSS), AM is ready for use [22,23,24,25]. There are various preparation methods for AM that will be applied for skin therapy, including decellularization, lyophilization, and isolation of stem cells.

AM is rinsed with sterile phosphate-buffered saline (PBS) for 30 min, immersed in Roswell Park Memorial Institute (RPMI) 1640 medium, and a total of 2.5 × 10^6^ mononuclear cells are cultured on the upper surface of decellularized human amniotic membrane (hAM). After 15 min, the decellularized hAM is inverted, and the same number of cells is grown on the contralateral side. This process is repeated every 15 min to 1 h to facilitate even cell distribution. Decellularized hAM containing mononuclear cells are placed in Dulbecco’s modified low-glucose media supplemented with 10% fetal bovine serum and antibiotics (100 U/mL penicillin G and 1 mg/mL streptomycin) in a humidified incubator (37 °C, 5% CO_2_), and incubated in culture media for 4 days [26,27,28,29]. According to published studies [30,31], AM decellularization can be carried out using the enzymatic method, namely 0.05% trypsin/0.02% ethylenediaminetetraacetic acid (EDTA) administered for 20 min at 37 °C, washing with PBS, and incubation at 37 °C in culture media before sowing cells.

Lyophilization or freeze-drying is a preservation technique that comprises removing water from tissues through a process of sublimation [32]. Decellularized AM that is seeded with mononuclear cells can be lyophilized. Lyophilization is carried out at −80 °C for 48 h. The product is then sterilized using 10 KGy gamma radiation with a cobalt-60 radiation source. Lyophilized AM can be thawed before being used up to 24 °C [26,33,34].

hAM can be cultured to obtain human amnion epithelial cells (hAECs) and human amniotic membrane-derived mesenchymal stem cells (hAMSCs). AM is cut into small pieces and digested with 1 mg/mL collagenase type IV for 1 h. The cell suspension is centrifuged to produce hAMSCs. AECs are fetal epithelial cells with a lifespan of less than a few months. AMSCs have the same capacity to differentiate into specialized cells as adult stem cells, including osteocytes, chondrocytes, adipocytes, cardiomyocytes, myocytes, neurocytes, and hepatocytes [32]. hAMSCs are cultured in DMEM-F12 media supplemented with 10% fetal bovine serum, penicillin 100 U/mL, and streptomycin 100 ng/mL at 37 °C and 5% CO_2_. When hAECs and hAMSCs reach 90% confluence in two sections, 2.5 × 10^6^ cells are grown in 75 cm^2^ cell culture flasks in serum-free EpiLife medium for 24 h [35]. In prior studies [36,37,38], researchers have grown cells for 12 weeks and digested them with 0.25% trypsin containing 0.02% EDTA. Culture media is collected from each sample and used as conditioned media [2,39].

## 5. Applications of Amniotic Membrane on The Skin Regeneration

The skin is continually exposed to a wide range of trauma. In several documented dermatological cases, the AM has shown the ability to promote skin regeneration. The AM has a crucial function as a medicinal agent used to treat various skin problems. Examples of AM skin application effects are summarized in Table 1.

### 5.1. Wound Healing

**Figure 1:**
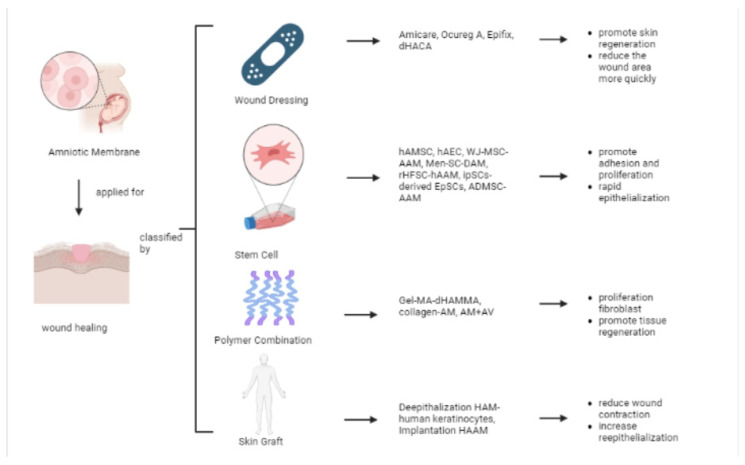
Examples of materials applied in skin wound healing.

Wound healing is a complex and dynamic interaction between various types of ECM cells, cytokines, and growth factors. Wound healing consists of several stages, including hemostasis, inflammation, cell migration and proliferation, wound contraction, and remodeling. Granulation tissue forms in open wounds and consists of macrophages, fibroblasts, immature collagen, blood vessels, and stroma [78]. Several studies, including in vitro, in vivo, and clinical testing, have studied the use of AMs for wound healing applications in the form of wound dressings, stem cells, polymer combinations, and skin grafts (Figure 1).

#### 5.1.1. Wound Dressing

Recent studies support the early adoption of advanced wound care products that hold promise for healing chronic wounds. The human placental membrane is a rich source of mesenchymal cells, collagen matrix, and growth factors, and it is capable of tissue regeneration and repair in wounds [75]. Therefore, several studies related to AM products have been conducted—including a three-site, prospective, randomized, control, parallel-group study to compare the healing effect of diabetic foot ulcer (DFU) treatment with weekly use of Apligraf^®^, EpiFix^®^, and collagen-alginate wound dressings [76]. The epidermal layer of Apligraf^®^ is composed of human keratinocytes and a well-differentiated stratum corneum; the dermal layer is made up of human fibroblasts in a bovine Type I collagen lattice [53]. Epifix^®^ is an allograft comprised of layers of the amniotic sac, such as the epithelial lining, amnion, and chorion, which contain vital biological substances such as collagen, connective tissue, cytokines, and growth factors [53]. After 1 week, EpiFix^®^ had reduced the wound area by 83.5% compared with a 53.1% reduction by Apligraf^®^. The median time to healing was significantly shorter with EpiFix^®^ (13 days) than with Apligraf^®^ (49 days) or standard care (49 days). These results demonstrate the clinical advantages and resource utilization of EpiFix^®^ (AM) compared with Apligraf^®^ or standard care in the treatment of DFUs [53,76].

Dried amnion (AmiCare^®^) and acellular amnion (OcuReg-A^®^) are new AM products, and Mepitel^®^ is the primary wound dressing [19]. Mepitel is a wound contact dressing that can be applied to a variety of acute wounds (such as skin tears, abrasions, and second-degree burns), chronic wounds, skin disorders (such as epidermolysis bullosa), and for the fixation of grafts [77]. Researchers compared these products and found negligible differences between dry and AAM regarding donor wound pain, healing time, and scar-ring. So, they concluded that dry AM is more practical and cost-effective to facilitate wound closure, but if autologous or allogeneic cells are planned to be transplanted into the wound, AAM is the optimal choice (Figure 1) [19].

Glat et al. evaluated dehydrated human amnion and chorion allograft (dHACA). They undertook a randomized comparison comparing dHACA for 12 weeks with one of the ear-liest and widely accepted skin tissue engineering substitutes (TESS) in the treatment of chronic DFU for 12 weeks. After 6 weeks, the median time to recovery was 24 days (95% confidence interval [CI] 18.9–29.2) for the dHACA group and 39 days (95% CI 36.4–41.9) for the TESS group. After 12 weeks, the median time to healing was 32 days (95% CI 22.3–41.0) for the dHACA group and 63 days (95% CI 54.1–72.6) for the TESS group. The per-centage of wounds healed at the end of the study (12 weeks) was 90% (27/30) in the dHACA group and 40% (12/30) in the TESS group. Therefore, it can be concluded that aseptically processed dHACA heals DFUs reliably, significantly faster, and significantly cheaper than TESS (Figure 1) [54].

The use of EpiFix^®^, a novel allograft derived from decellularized hAM, improved wound healing in a number of patients previously identified as unresponsive to conventional methods and biological tissue applications [43]. The decellularized hAM allograft was applied according to the manufacturer’s instructions to wounds made by sharp dis-section; there was no contraindication to the use of allograft placement. This material is also well tolerated by patients. In one case, direct application of decellularized hAM to an exposed tendon without scar tissue formation resulted in tendon slippage and wound closure. Wound sites still healed without long-term recurrence (Figure 1) [43].

The results of AM preparation in the form of lyophilization were also evaluated for the feasibility of developing soluble amniotic membrane extract (AME) as a potential wound-healing substrate. In a cell migration study performed 24 h after injury, AME cells (1.7 µg/mL) increased wound closure by 54.9% compared to controls. Histological analysis of the AME-treated wound sites 36 days after injury showed a well-developed epidermal basal cell layer, whereas the untreated control showed excessive proliferation, damaged epidermis, and aggregated collagen pools in regenerated skin [55].

In another study, researchers developed a dermal equivalent (DE) using bone marrow stromal cells (BM-SCs) expressing TGF-β3 and decellularized hAM and demonstrated TGF-β3 expression during the early stages of wound healing [50]. Histological evaluation showed that transfused BM-SCs could reduce cell retention and recruitment and reduce angiogenesis and uniform parallel collagen formation during the early stages of wound healing. The Manchester Scar Scale (MSS) score indicated that TGF-β3-secreting cells significantly improved the appearance of healed skin and reduced scarring. These results suggest that the transient secretion of TGF-β3 during the early healing phase can improve the appearance of scars without affecting the wound healing process [50]. It is well reported that TGF-β3 functions as a wound-healing promoting factor, and thereby if in excess, it may lead to overhealing outcomes, such as hypertrophic scarring and keloid.

Song et al. applied decellularized hAM in mice to correct full-thickness skin defects. Decellularized hAM is a biocompatible wound dressing with excellent wound healing. It mainly plays a role during the proliferative phase and increases the expression level of vascular endothelial growth factor (VEGF), which promotes skin regeneration through angiogenesis involved in remodeling such as ECM contraction and remodeling—contributing to increased αSMA secretion (Figure 1). In addition, it reduces wound inflammation in the first days after surgery, thereby reducing scar tissue by modulating excessive levels of transforming growth factor beta-1 (TGF-β1), which acts during the primary inflammatory stage and induces VEGF and αSMA biosynthesis [29].

#### 5.1.2. Stem Cell

Among cell-based therapies, the use of stem cells for wound healing potential has been widely recognized. Normal wound healing requires coordinated communication between cells, growth factors, and ECM proteins. Activated stem cells are critical in this process as they enable a coordinated repair response [78,79].

Based on in vitro experiments, hAMSCs secrete proteins that promote epidermal growth and development. Media conditioned by hAMSCs (hAMSC-CM) and by hAECs (hAEC-CM) induce keratinocyte migration, but hAEC-CM are slightly stronger than hAMSC-CM [35]. However, hAMSCs are genetically stable for large-scale clinical applications and product development. hAMSC-CM is rich in proteins that regulate wound healing, angiogenesis, cell differentiation, the immune response, and cell motility. In vivo research showed that both hAECs and hAMSCs have anti-inflammatory and tissue re-modeling properties and are effective in healing acute and chronic skin wounds. hAMSC-CM and hAEC-CM contain proteins that encourage wound healing both in vitro and in vivo. These include lysyl-oxidase-2 (LOXL2), which promotes wound healing by activating the c-Jun N-terminal kinase (JNK) signaling pathway [35].

Researchers performed Wharton’s jelly mesenchymal stem cells (WJ-MSC) and der-mal fibroblast (DF) cultures on three-dimensional (3D) AAM and used it to treat chronic ulcer wounds in five patients diagnosed with type II diabetes [51]. The results showed con-traction and wound healing of the skin due to the rapid epithelialization of chronic diabetic ulcers. The cell therapy technique in this research healed wounds within 2 weeks—much faster than the >1 year that would normally be required (Figure 1) [52].

Kim, S.S. et al. found that autologous MSCs loaded into transplanted hAM promoted wound healing and epithelial differentiation at the wound site. hAM and autologous MSCs used in this trial are approved for clinical use in humans and could improve wound healing [22]. In another study, researchers examined the transplantation of menstrual blood-derived stem cells (MenSCs) through decellularized hAM to improve wound healing [47]. They used decellularized hAM as a platform for stem cell transplantation into damaged skin. The best recovery results were obtained in the MenSC–decellularized hAM group. MenSCs may be potential candidates for cell therapy to enhance wound healing [74]. The decellularization process affects AM formation. After decellularization by physical and chemical methods, the structure of hAAM resembles the basement membrane of the skin and cornea. It provides sufficient 3D space for cell growth and promotes adhesion and proliferation (Figure 1) [74].

In another study, researchers labeled >90% of rat hair follicle stem cells (rHFSC) with 5-ethynyl-2-deoxyuridine (EdU) [46]. They then cultured the labeled cells with hAAM for 5 days and implanted them onto the wound surfaces of naked mice. After 1 week of treatment, hematoxylin and eosin staining and CD31 immunohistochemistry showed that newly formed blood vessels were more prominent than in controls. They confirmed the formation of accessory skin organs under induction in an in vitro microenvironment. In particular, rHFSC–hAAM treatment produced the most hair follicles [46].

AM could also contribute to the induced pluripotent stem cells (iPSC) system. Based on scanning electron microscopy, cells cultured in hAAM for 4 days had similar morphology to epithelial stem cell (EpSC) derivatives of iPSCs cultured in hAAM, indirectly confirming the cytocompatibility of AM as a scaffold for skin replacement. In vivo research showed that wounds treated with hAAM-containing cells had a significantly shorter healing time than wounds treated with hAAM and Vaseline; therefore, treatment with hAAM–cell complexes accelerated wound healing and the structure of newly formed hair follicles. In addition, green fluorescent protein (GFP) was detected in the epidermis and newly formed wound hair follicles treated with hAAM and cell complexes. These results showed that the skin substitute consisting of hAAM-derived EpSCs and iPSCs promotes wound healing and is involved in the formation of skin appendages. Therefore, iPSC-derived EpSCs, which are used as germ cells to make skin substitutes, are potential candidates for repairing skin defects, reconstructing skin appendages, and restoring skin function [44].

Researchers have also examined the phenomenon of hair follicle development during wound healing by using adipose-derived mesenchymal stem cell (ADMSC)–AAM compo-sites to improve full-thickness skin disease [45]. The adipose stem cells adhered tightly to the AAM surface and proliferated well for 24 h after culture. By day 7, the cells had coalesced into spots and covered the AAM surface. Hair follicles developed during the full-thickness skin defect process using ADMSC–AAM composites. These findings could be clinically important in the treatment of full-thickness skin defects using skin tissue engineering [45].

#### 5.1.3. Polymer Combination

Q. Zhang et al. studied the use of decellularized hAM and gelatine to synthesize and characterize photo-crossed bicomponent-network (BCN) hydrogel, namely GelMA-dHAMMA. This composite material has excellent mechanical properties of GelMA and inherits the biological activity of decellularized hAM. GelMA-dHAMMA itself has a natural ECM component and thus better simulates the ECM microenvironment, providing a dynamic environment for fibroblast proliferation and deposition, supporting fibroblast proliferation and differentiation, and thereby promoting tissue regeneration (Figure 1) Because de-cellularized hAM is rich in collagen, it reacts with methacrylic anhydride (MA) via the amino groups of collagen in decellularized hAM collagen and methacryloyl in the grafts to form dHAMMA [49].

Researchers have also compared amnion hydrogel and amnion powder for full-thickness wounds. These products resulted in the fastest rate of wound closure, mainly through new epithelialization, reduced contraction, and elimination of immune resistance. This observation is supported by histological analysis: this treatment promoted rapid healing of these full-thickness wounds, forming a mature epidermis and dermis with a composition similar to healthy skin [33]. The newly developed collagen—AM combination can act as a skin substitute, inhibiting inflammatory reactions and promoting wound healing (Figure 1). This approach led to faster healing, and histological analysis revealed fewer inflammatory cells and more hyperplastic fibroblasts. Four weeks after surgery, a significant amount of new collagen had formed in the AM-dehydrothermal group [34].

Rahman et al. combined aloe vera (AV) and AM in the form of gel preparations. Based on morphological analysis, this preparation accelerated wound contraction due to gel epithelialization in vivo. Microscopic observation of the processed AM+AV tissue sections made it possible to assess the efficacy of the formulated gel in terms of epidermal and dermal formation and epidermal thickness. The AM+AV group had a higher degree of inflammatory epithelialization. The wounds treated with AM, AV, and AM+AV showed more blood vessels, especially newly formed small vessels. In vitro and in vivo data clearly show that AM+AV extract significantly improved burn wound healing [42].

#### 5.1.4. Skin Graft

Clinical testing on foot ulcers has proved that hAM induces human keratinocytes in the de-epithelialization process, meaning that the amniotic stroma plays an equivalent role in the dermis [40]. Enzymatic de-epithelialization of hAM has been validated and maintains the integrity of the basement membrane. The addition of the reconstructed epidermis to de-epithelialized AM allows for rapid and frequent reepithelialization of the wound, especially in foot ulcers [40]. In another study, researchers found that the human acellular amniotic membrane (hAAM) implantation model reduced postoperative bleeding and thinning scabs in the promoted wound healing, particularly in repairing full-thickness defects in the lower third of the nose in humans [73]. This may be related to its role in hemostasis and increased granulation tissue formation within 24 h. This clinical study confirmed that hAAM transplantation is feasible and effective for the reconstruction of the lower third of the nose. Faster wound healing, reduction of postoperative complications and easy of use make the hAAM-based approach a promising procedure for the treatment of skin defects. It has great potential for widespread use in skin restoration [73].

In in vivo testing using pig skin, compared with another skin substitute (IntegraVR) as a control, multilayer hAM reduced wound contraction, increased reepithelialization, and provided better quality scar tissue (based on histology) in wounds (Figure 1). In full-thickness wounds, the scar quality of the wound decreased, and alpha-smooth muscle actin (αSMA) supported continuous scar maturation [41].

### 5.2. Burn Injury

**Figure 2:**
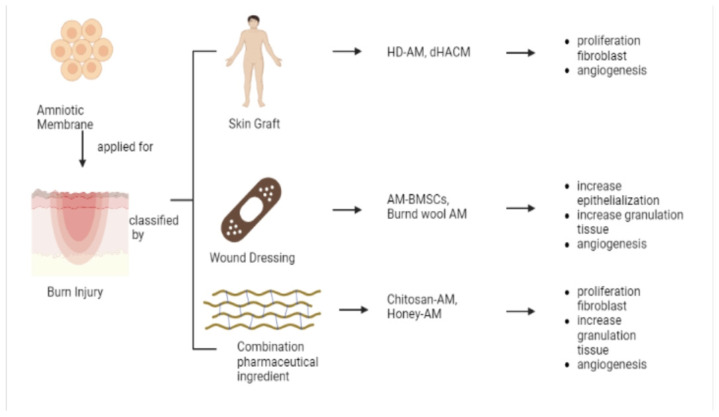
Examples of materials applied in skin burn injury.

Burns cause significant fluid loss and extensive tissue damage, causing deep scars and impairing vital skin functions. Therefore, burns are difficult to heal [79]. The risk of infection and sepsis, scar tissue, and joint problems are some of the complications [74]. The AM can give great coverage to burn sites in preparation for skin grafts, wound dressings, and pharmaceutical ingredient combinations (Figure 2).

#### 5.2.1. Skin Graft

AM has a special capacity to adhere firmly to the injured area [11]. Dehydrated hu-man amnion chorion membrane (dHACM) is an alternative burn graft that improves wound healing and reduces scar tissue formation. Treatment with dHACM resulted in faster healing than split-thickness skin grafts (STSGs). Scars showed partial thickness but less scarring in deep, full burns compared with STSGs [56]. Similar research using hy-perdry-AM (HD-AM) encouraged good initial granulation growth [59]. Cytokines and scaffold residues of HD-AM encourage inflammatory cell infiltration during the early stages of granulation tissue formation with fibroblast proliferation and angiogenesis (Figure 2). At the same time, neutrophil migration can be enhanced, and defense mechanisms against foreign substance invasion can be strengthened. Stronger HD-AM induction during the early inflammatory phase allows for a faster and smoother transition to the proliferative phase. Therefore, HD-AM is useful as a wound dressing for total skin excision after third-degree burns and may be a new therapeutic technique to improve the survival rate of severe burn patients [59]. Furthermore, when researchers compared a control treatment with STSGs using AM, the control group had a higher pain score [60]. The moist environment within the occlusive dressing facilitates adequate removal of the wound dressing with minimal damage to the newly formed smooth epithelial layer. AM as an alternative dress-ing for STSGs offers significant benefits by increasing patient comfort while reducing pain and accelerating the wound healing process [60].

Researchers have compared AM and Matiderm skin substitutes in the therapeutic management of a pig model with third-degree burns [24]. At the clinical level, the presence of AM reduced involution scars and improved cosmetic results. Furthermore, the pinch test showed more supple skin with the presence of AM, which could be explained by the presence of well-formed elastin fibers that were only seen when AM was attached. Overall, AM stimulated the synthesis of elastin fibers, induced a network of elastin fibers, and completed the skin replacement of the wound [24].

#### 5.2.2. Wound Dressing

In vivo testing using mice showed that using AM in humans and burned wool had a significant therapeutic effect on deep, second-degree burns [58]. Pathology specimens showed increased epithelialization, granulation tissue, and angiogenesis and decreased inflammatory cells and macrophages at days 7, 14, and 21 (Figure 2). Burned wool had a greater impact on the establishment of granulation tissue and epithelium than SSD ointment and the untreated negative control group [58]. Other results were: (1) wound healing in Group II animals was about 2.7 times slower than in Group I animals. (2) The use of decellularized AM to cover burns increased the healing rate by 2.5 times. (3) The use of decellularized AM with bone marrow-derived stem cells (BMSCs) increased the wound healing rate four-fold. (4) Bioactive wound dressing (BAWD) increased the wound healing rate by about four-fold, similar to Group IV animals. Inflammation did not occur when using decellularized hAM. This is clearly related to its anti-inflammatory and expression barrier proper-ties. Lyophilized BMSCs retain unique paracrine factors and, when combined with decellularized and lyophilized hAM, encourage clinical wound healing and wound epithelialization [26].

#### 5.2.3. Pharmaceutical Ingredient Combination

Combining AM and other pharmaceutical ingredients also plays a role in burn healing. AME–chitosan gel preparations led to faster epidermal and dermal regeneration, an increase in granulation tissue and fibroblast proliferation and improved capillary blood formation with the development of collagen (Figure 2) [61]. Therefore, AME–chitosan gel can be considered a simple and easy-to-apply biological bandage for all types of superficial burns without any side effects. Another research by chitosan/collagen-AM hydrogel was generated by adding a solution containing AM and collagen powder to the gelling agent (CMCNa) and was utilized in conjunction with or alone from a chitosan/collagen-blended amniotic membrane. This biocompatible hydrogel resulted in rapid wound healing in a rat model, with complete re-epithelialization and wound contraction [80]. Rad et al. [61] found that honey-infused AM is a convenient and effective topical agent for burn healing and secondary burn reconstruction. The main underlying etiology by which the placental membrane reduces polymorphonuclear leucocyte migration is unknown, but it may act through the initiation of apoptosis [62]. The placental membrane can encourage the formation of granulation tissue through fibroblast proliferation and angiogenesis (Figure 2). Honey contains low concentrations of glycosaminoglycans and proteoglycans that stimulate the synthesis of high-quality collagen and increase the rate of cross-linking between fibers. Honey also encourages rapid wound epithelialization [62].

### 5.3. Skin Tissue Engineering (TE)

**Figure 3:**
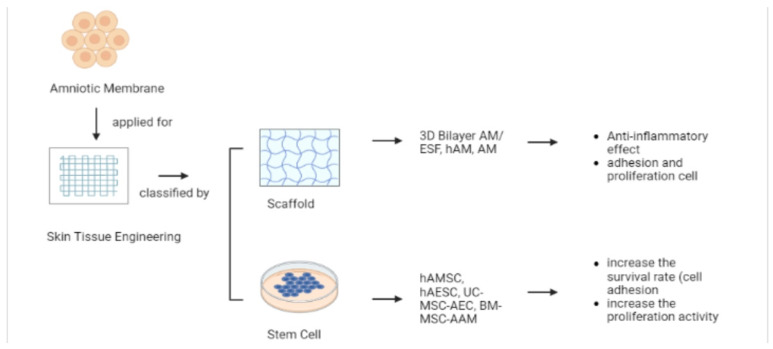
Examples of materials applied in skin tissue engineering.

Tissue engineering applications include the study of cell biology, wound healing, testing systems for therapy, and the formation of living tissue for drug delivery [81]. The human amniotic membrane is a prospective scaffold and stem cell for skin tissue engineering (Figure 3).

#### 5.3.1. Scaffold

One form of tissue engineering application is in the form of a scaffold. The greatest materials for healing, preserving, and enhancing tissue function are scaffolds. They perform a unique function in tissue repair and, more critically, regeneration by providing a proper substrate that enables the essential supply of several elements related to cell survival, proliferation, and differentiation [82]. The amniotic membrane, the innermost layer of the fetal (or placental) membrane, has been utilized as an allograft to promote the repair and regeneration of corneal epithelial and stromal cells and tissues with little inflammation and scarring. AM provides a stable basement membrane for cell culture, expresses anti-immunogenic and anti-inflammatory agents and shows good results in the treatment of deformed wounds [27,83]. The application of hAM accelerates the wound-healing process by closing the wound. The anti-inflammatory action of hAM shortens the duration of the early healing phase and promotes the early onset of collagen fibrillogenesis, such as in the remodeling phase characterized by the replacement of type III (immature) collagen with type I (mature) collagen. Researchers have shown that hAM plays a protective role in the wound area and provides a favorable microenvironment for skin repair [23]. In another study, AM significantly increased valve viability, decreased the number of polymorphonuclear leucocytes, and increased the proliferation of capillaries and the number of blood vessels in the experimental group [20]. Although the exact mechanism of this effect is unknown, it is possible that the anti-inflammatory effect of AM reduces the activation and leucocyte proliferation in the acute phase (Figure 3). AM also prevents cell degradation by free radicals in the acute phase by preventing lipid and protein peroxidation. Therefore, AM can be used for randomized or extended axial flap surgery. AM can improve the survival rate of ischemic skin flaps [20].

Decellularized hAM has also been used in a 3D bilayer scaffold using viscoelastic electrospun nanofiber silk fibroin (ESF) [63]. The 3D bilayer made from the AM–ESF scaffold was immersed in ethanol to induce the transformation and to produce a dense and inseparable bilayer. The 3D bilayer AM–ESF scaffold had markedly increased mechanical properties compared with AM alone. Both AM and AM–ESF had corresponding variations in cell adhesion, and there was no cytotoxicity against ADMSCs. These cells showed increased expression of two main pro-angiogenic factors, VEGFα and bFGF, when cultured with AM/ESF for 7 days compared with AM alone. The results revealed that AM–ESF scaffolds with autologous ADMSCs showed excellent cell adhesion and proliferation, and the production of growth factors served as potential applications in the clinical setting of skin regeneration (Figure 3) [63].

#### 5.3.2. Stem Cell

Due to their propensity for self-renewal and differentiation into specialized cell types, stem cells have attracted attention in skin tissue engineering and regenerative medicine. AMs can also be applied to the skin in the form of stem cells [84]. hAMSCs and hAECs from the AM can be isolated and display fibroblastic and cobblestone-shaped epithelial morphology, respectively. Both hAMSCs and hAECs are positive for CD73, CD90, and CD105 and negative for CD34 and HLA-DR. Reverse transcription–polymerase chain reaction (RT-PCR) revealed that hAMSCs express the stem cell markers Nanog and c-MYC, and the keratinocyte markers K19, β1 integrin, and K8; hAECs express the stem cell markers KLF4 and c-MYC, and the keratinocyte markers K19, β1 integrin, K5, and K8. Finally, histological analysis showed that tissue-engineered skin is structurally similar to normal skin [36].

The placenta is a potential source for cells to develop into skin-like cells. AECs and UC-MSCs could be induced in vitro to differentiate into keratinocytes and fibroblasts. After induction, morphological changes were detected microscopically. The researchers assessed the potential for further differentiation by using immunostaining analysis and RT-PCR. AECs express cytokeratin and E-cadherin, whereas UC-MSCs express fibroblast-specific markers. AECs that differentiate into keratinocyte-like cells show positive expression of keratinocyte-specific cytokeratin, involucrin, and loricrin. UC-MSCs that differentiate into dermal fibroblast-like cells show expression of type 3 collagen, desmin, FGF-7, fibroblast activation protein alpha, procollagen-1, and vimentin [65].

RSF treatment with a BM-MSC–AAM bioscaffold significantly increased survival. Furthermore, the application of a BM-MSC–AAM bioscaffold was more effective than AAM or saline alone. A possible mechanism for AAM is to act like an ECM. The basic part of AM is used for cell growth and plays a major role in cell adhesion during cell seeding protocols (Figure 3) [64]. Another study showed that a BM-MSC–AAM bioscaffold increased the total number of mast cells and encouraged capillary growth at the transient site of RSF in mice. BM-MSCs can affect the speed and quality of wound healing. The combination of these two features separately can increase the efficacy of both treatments in flap ischemia and improve the prognosis after flap implantation. Using AAM and MSCs, researchers investigated the effect of AAM on mast cells during ischemia in the dorsal RSF [18]. There was an increase in the number of type 2 mast cells compared with controls. AAM has an important effect in reducing the number of type 3 mast cells, converting type 2 mast cells into type 3 mast cells [18].

### 5.4. Other Applications

**Figure 4:**
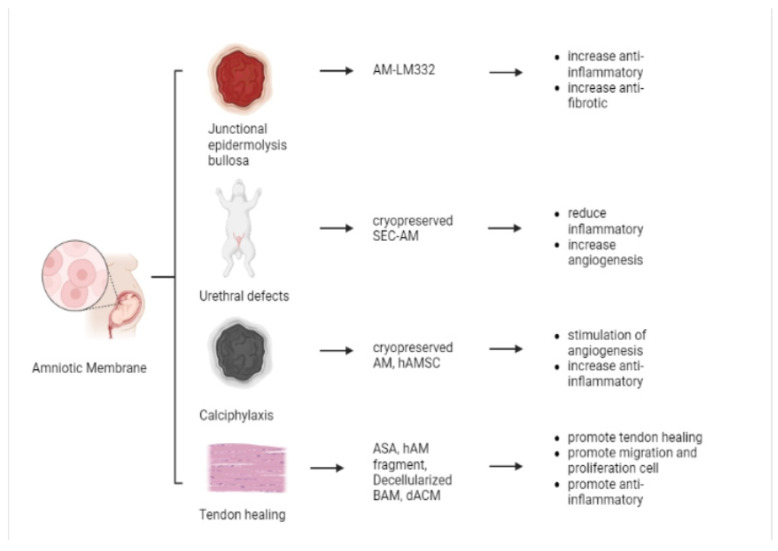
Examples of materials applied in the skin for JEB, urethral defects, calciphylaxis and tendon healing.

Another application is applying AMs to the skin in several diseases, including junctional epidermolysis bullosa (JEB), urethral defects, calciphylaxis and tendon healing (Figure 4).

#### 5.4.1. Junctional Epidermolysis Bullosa (JEB)

Junctional epidermolysis bullosa (JEB) is a clinically and genetically heterogeneous skin fragility disorder generally caused by mutations in the gene encoding laminin 332, an epithelial isoform. AM contains antiangiogenic and antiinflammatory compounds having the ability to reduce eye damage following injury, promote ocular surface repair, and possess tectonic properties (Figure 4) [67]. Researchers have evaluated the therapeutic potential of an ophthalmic formulation in hAM in corneal lesions [67]. They isolated the AM from donor placenta and confirmed laminin 332 expression. Their AM eye preparations could restore keratinocyte adhesion in vitro. Accordingly, AM eye drops may be an effective, non-invasive, and simple therapy for treating corneal lesions in patients with JEB and possibly other forms of elevated EB [67].

#### 5.4.2. Urethral Defects

Urethral defects caused by trauma to the urethra, congenital malformations, and tumors are common urological diseases [85]. AM can reduce cryopreservation damage to SEC cell activity and allow more EpSCs to survive. This combination also masks the weak mechanical properties of SEC, so it is useful for surgical procedures. Furthermore, researchers pre-vascularized inguinal rabbit sections for 2 weeks and found that cryo-SEC-AM reduced inflammatory cell infiltration and exhibited greater angiogenesis (Figure 4). An angiogenic graft is then used to repair the urethral defect in rabbits. Complete epithelialization and angiogenesis in the cryo-SEC-AM group were confirmed histologically [68].

#### 5.4.3. Calciphylaxis

Calciphylaxis is a rare disease characterized by skin ulceration and necrosis due to vascular calcification of small- and medium-sized blood vessels in the skin and subcutaneous tissue. This is especially true in patients with advanced chronic kidney disease, and this condition can lead to fatal complications. Researchers have found that patients experienced a decrease in pain as measured by a visual analog scale after treatment with AM. The treatment contributed to ulcer epithelialization despite colonization with methicillin-sensitive *Staphylococcus aureus*. AMs increased the healing of unresponsive ulcers, restored skin integrity, and reduced the pain experienced by patients [69].

In a preclinical study on calcification disease, researchers administered hAMSCs via local intramuscular injection, intravenous injection, and application of external supernatant to wounds [70]. During up to 15 months of follow-up, there were improved blood-based markers of bone and mineral metabolism, softer skin regeneration tissue, and improved peripheral blood mononuclear cell profile. Skin biopsy after 1 month of treatment was revascularized by mature uncalcified vessels in the dermis and de-epithelialized after 20 months, restoring the integrity of the damaged site. There are no injections or side effects associated with topical medications. In conclusion, hAMSC treatment promotes skin and soft tissue repair. This result indicates inhibition of vascular calcification, stimulation of angiogenesis, myogenesis, and anti-inflammatory activity (Figure 4) [70].

#### 5.4.4. Tendon Healing

The tendon healing process comprises three major phases: inflammatory, proliferative, and scarring. During the time of inflammation, a hematoma releases chemoactive substances and vasodilators, which induce the development of new blood vessels. Collagen synthesis is high during the proliferative phase. However, Type III collagen predominates. During the scar phase, the ruptured end of the tendon is reconnected, and Type I collagen begins to settle in parallel to the tendon axis [86]. Semipermeable, nonvascular, and abundant in cytokines, the amniotic membrane is an appropriate biomaterial for inhibiting tendon attachment. At the tendon healing site, HAM wrap generates a “tunnel” similar to the “tendon sheath.” The advantage of ultrasonography is its capacity to offer real-time, dynamic images of a moving tendon. After tendon injury and healing, HAM decreases the serum level of proinflammatory mediators IL-6 and TGF-β1, which is believed to have an immunomodulatory effect. After tendon wrapping, a lower level of the immunomodulatory cytokine TGF-β1 may represent the proinflammatory condition of the cytokine network. At two to six weeks post-operatively, the levels of serum inflammatory biological markers dropped in the majority of HAM cases, whereas they increased in controls. Infection and immunological rejection were not observed in HAM wrap instances. HAM wrap over the tendon repair site resulted in faster function and qualitatively superior tendon healing as measured by ultrasonography, with a reduction in the biologic reaction [87].

Another study was to investigate the efficacy of biomaterial on the inflammatory response and organization of collagen fibers at various phases of the Achilles tendon’s acute injury healing process. At the beginning of the healing phase, the inflammatory response in groups T and I was comparable, but the number of inflammatory cells was significantly lower in specimens from the T group. At 3 days, an intensive neutrophilic inflammatory infiltration and disruption of the pre-existing fibers were observed in groups I and T, confirming that the damage was effectively generated in the human participants of this investigation. Application of a human AM fragment around the injured tendon did not boost the inflammatory response in the lesion area, confirming the minimal immunogenicity of hAM observed in prior investigations [72].

Combining a collagen–glycosaminoglycan scaffold previously developed for tissue regeneration and a matrix material (hyaluronic acid and AM) increased healing and reduced scarring [77]. In the study, the AM matrix–containing scaffold significantly improved mechanical properties, and tendon cells in this scaffold maintained their metabolic activity even when the media included the pro-inflammatory cytokine interleukin-1 beta. A collagen scaffold containing hyaluronic acid or AM can also attenuate gene expression (TNF-α, collagen I, and MMP-3), which is related to the inflammatory response in normal tendon healing and modifies the inflammatory response of tendon cells [77]. TNF-α, which collaborates with IL-1 in tendon repair, is increased in adult tendon healing and downregulated in CS and AM scaffolds. Within the CG scaffold platform, these fetal ECM components may preserve their anti-inflammatory capabilities. This protein is significantly downregulated in cells on HA, AM, and CG scaffolds under circumstances of high inflammatory media [77].

In an ongoing effort to construct a rodent model of chronic Achilles tendinopathy, rats were administered the anticoagulant human amniotic suspension allograft (ASA). Compared to mice treated with saline, treatment with ASA increased tissue healing after 14 days and decreased the presence of collagen type III in the tendon. The ASA treatment was well tolerated, and this trial offered significant evidence about the retention of ASA in tendon tissue following treatment. Two weeks after collagenase type I injection, the administration of ASA resulted in considerable improvements in fiber alignment, ECM restoration, and physiologic cellularity [71].

Another In vitro and in vivo studies have demonstrated that amniotic membrane (AM) promotes anti-inflammatory, scarless wound healing (Figure 4). This tissue is biologically active and includes a multitude of growth factors/cytokines that are required for fetal tissue development and wound healing. In this paper, AM matrix was introduced into 3D collagen biomaterial for tendon healing applications using two methods: bulk incorporation to build a porous collagen-AM scaffold or as a membrane wrap encircling a collagen-glycosaminoglycan scaffold to form a core-shell composite. In response to an inflammatory media challenge, evaluated the metabolic activity and immunomodulatory response of human mesenchymal stem cells within these scaffolds. Expression profiles of the immunomodulatory genes IL-6 and IL-8 indicate that whereas MSCs exhibit a response to media challenge, MSCs within AM-functionalized collagen scaffolds have a variable response in the early stages following an inflammatory challenge. These findings, along with the possibility of using the amniotic membrane as a wrap to increase the mechanical performance of the resultant composite while maintaining the beneficial immunomodulatory effect of the AM matrix, suggest a viable route for incorporating amniotic membrane matrix into three-dimensional biomaterials to enhance tendon regeneration potential [75].

Proposed tendon-healing processes are a combination of extrinsic and intrinsic mechanisms. The amniotic membrane inhibits the healing mechanism with anti-scarring and anti-inflammatory actions while promoting the intrinsic healing mechanism with the unique extracellular matrix components. Less inflammation, a more mature arrangement of fibroblasts and collagen fibers, and improved biomechanical qualities were observed in the early healing phase as a result of this study. Amniotic membrane promoted the maturation of the histological organization of fibroblasts and collagen fibers and improved the properties of lacerated tendons during the early stages of healing. Biomechanically, the treatment group’s tendon modulus at four weeks was substantially greater than the control group’s (*p* < 0.05) [76].

Injured diabetic tendons exhibited a considerable delay in healing, as evidenced by decreased biomechanical characteristics and cell density at the location of injury 28 days after injury. It has been observed that placental-derived grafts include several growth factors and cytokines believed to promote tendon healing (Figure 4). It has been demonstrated that dehydrated human amnion/chorion membranes (dACM) promote cell proliferation and migration in a range of cell types (Figure 4). dACM-wrapped tendons may be associated with a quicker transition from the inflammatory to the repair phase of tendon healing [73].

## 6. Author’s Perspective

AM is an abundant tissue and is easily obtained after delivery because it is extracted from the human placenta. AM is a valuable material for tissue repair and a viable alternative to synthetic or natural scaffolds. It contains many proteins and growth factors, including EGF, bFGF, TGF-β, and KGF, and has interesting properties and structures [83]. AM contains two distinct cell types, AECs and AM-MSC, with characteristics of stem cells [8,32,85]. The unlimited availability of AM, high-efficiency recovery, non-invasive and safe procedures, minimal ethical and legal concerns (so there are no problems associated with its use), and the low immunogenicity of the derived cells make it an alternative source of stem cells. AMs can be applied in skin therapies such as wound healing, burns, tissue engineering, JEB, urethral defect, calciphylaxis, and tendon healing. The preparation of extracts or powders from native AMs and their combination with biodegradable polymers holds promise for tissue engineering with AM-based constructs that exhibit suitable properties for various applications. The weakness of using AMs is the lack of research on the molecular mechanisms, stability, and long-term effects. Therefore, additional research is needed to improve the stability of AM in cell proliferation and the efficiency of differentiation and cell-based therapies that use skin progenitor cells to promote wound healing so that AM can be used for long-term skin therapy.

## 7. Conclusions

AM, originating from the human placenta, has benefits as a therapeutic agent for skin conditions. Research regarding the formulation and application of AM in skin therapy is still very limited. Researchers have reported that AM can be applied in several forms: decellularized, lyophilized, cryopreserved, and as a source of stem cells. Applications of AM in skin therapy include wound healing, burns, skin tissue engineering, JEB, urethral defect, calciphylaxis and tendon healing. Additional clinical research on AMs should lead to a more standardized protocol for its use and further confirm the safety of AM applications.

## Figures and Tables

**Table 1:** The Application of Amniotic Membrane on the Skin Regeneration.

No	Application	Preparation Method	Experimental Settings (Target Tissue/Cells)	Ref.
1	Wound Healing	HAM de-epithelialization	In vitro (human epidermal keratinocytes)	[40]
		Lyophilized amnion membrane powder, Amnion hydrogel	In vivo (porcine skin)	[33]
		Fresh HAM	In vivo (porcine skin)	[41]
		Dried amniotic membranes	In vitro (human keratinocytes (HaCaT), human fibroblasts (HFF1 cell line); in vivo (Wistar rats))	[42]
		Dried AM	In vivo (porcine skin)	[34]
		Dehydrated human amniotic membrane allografts	Clinical exam (4 patients)	[43]
		HAM de-epithelialization	In vivo (rabbit)	[22]
		Dried human acellular amniotic membrane (hAAM)	In Vitro (NS)	[44]
		Dried amniotic membrane	Clinical exams (20 patients)	[19]
		Decellularized HAM	In vivo (mice)	[45]
		Dried amniotic membrane	In vivo (mice)	[46]
		Freeze Dried HAM	Clinical exams	[47]
		Conditioned media (CM) of hAMSC	In vitro (human mesenchymal stem cell adipogenic differentiation medium); in vivo (mice)	[2]
		Decellularized human amniotic membrane	In vivo (Wistar rats)	[48]
		Decellularization of amniotic membrane, AM Hydrogel	In vitro (human fibroblasts); In vivo (rabbits)	[49]
		Human dehydrated amniotic membrane	In vitro (TGF-β3); in vivo (Wistar rats)	[50]
		Conditioned Media of hAMSC and hAEC	In vitro (lysyl oxidase-like 2 (LOXL2); in vivo (mice))	[35]
		Decellularized Bovine Amniotic membrane	In vitro (human foreskin fibroblasts; in vivo (mice))	[51]
		Decellularized Amniotic membrane	Clinical exams (5 patients)	[52]
		Dehydrated human amnion/chorion membrane (dHACM)	Clinical exams (65 patients)	[53]
		Dehydrated human amnion and chorion allograft (dHACA)	Clinical exams (72 patients)	[54]
		Decellularized human amniotic membrane	In vivo (VEGF, α-SMA, TGF-β1; rats)	[29]
		Lyophilized Bovine AM	In vitro (human foreskin fibroblasts); in vivo (rabbits)	[55]
2	Burn Injury	Cryopreserved HAM	In vivo (porcine skin; clinical exam)	[24]
		Decellularized and lyophilized human amniotic membrane	In vivo (rats)	[26]
		Dehydrated human amnion chorion membranes (dHACM)	Clinical exam (30 patients)	[56]
		Dried AM	Clinical exam (35 patients)	[57]
		Fresh HAM	In vivo (male rats)	[58]
		Hyperdry human amniotic membrane	In vivo (mice)	[59]
		Dried amniotic membrane	Clinical exams (42 patients)	[60]
		Cryopreserved HAM	In vivo (rats)	[61]
		Fresh AM	In vivo (rats)	[62]
3	Tissue engineering (TE) skin	Decellularization of amniotic membrane	In vitro (NS)	[63]
		Fresh AM	In vivo (rats)	[64]
		Amniotic epithelial cells (AECs)	In vitro	[65]
		Fresh HAM	In vivo (Wistar rats)	[23]
		Fresh HAM	Clinical exams (30 patients)	[66]
		De-epithelialization of the hAM	In vitro (keratinocytes and fibroblasts)	[27]
		Fresh AM	In vivo (Wistar rats)	[18]
		Fresh AM	In vivo (Wistar rats)	[20]
		Isolation of human amniotic mesenchymal stem cells and human amniotic epithelial cells	In vitro (phycoerythrinor fluorescein isothiocyanate-conjugated monoclonal antibodies against human CD34, CD73, CD90, CD105, and HLA-DR)	[36]
4	Junctional Epidermolysis Bullosa	AM eye drop	Clinical exams (NS)	[67]
5	Urethral Defects	Decellularized Amniotic Membrane	In vitro (NS); in vivo (rabbits)	[68]
6	Calciphylaxis	Cryopreserved human amniotic membrane	Clinical exams (1 patient)	[69]
		Isolation of human amnion-derived mesenchymal stem cells (hAMSC)	Preclinical exams (mice and rats)	[70]
7	Tendon healing	Amniotic suspension allograft	Preclinical exams (rats)	[71]
		Fresh AM	In vivo (Wistar rats)	[72]
		Dehydrated human amnion/chorion membranes (dACMs)	In vivo (rats)	[73]
		Fresh AM	Clinical exams (17 patients)	[74]
		Lyophilized AM	In vitro (interleukin 6 (IL-6) and interleukin 8 (IL-8))	[75]
		Decellularized bovine amniotic membrane	In vivo (rabbit)	[76]
		Decellularized AM	In vitro (TGF-β1)	[77]

## Data Availability

All data included in the article.

## Abbreviations

AM	Amniotic membrane
ECM	Extracellular matrix
b-FGF	Basic fibroblast growth factor
EGF	Epidermal growth factor
KGF	Keratocyte growth factor
HGF	Hepatocyte growth factor
HIV	Human immunodeficiency virus
HCV	Hepatitis C Virus
HBs	hepatitis b virus
PBS	phosphate buffer saline
RPMI	Roswell Park Memorial Institute
EDTA	ethylenediaminetetraacetic acid
NaCl	natrium chloride
AEC	amnion epithelial cells
AMSC	amniotic membrane mesenchymal stem cell
HAMSCs	human amniotic mesenchymal stem cells
DMEM-F12	Dulbecco’s modified eagle medium/nutrient mixture F-12
hAECs	human amniotic epithelial stem cells
HAM	human amniotic membrane
hAAM	human acellular amniotic membrane
CM	conditioned media
dHACM	dehydrated human amnion/chorion membrane
dHACA	dehydrated human amnion and chorion allograft
dACM	dehydrated human amnion/chorion membranes
VEGF	vascular endothelial growth factor
α-SMA	smooth muscle alpha-actin
TGF-β1	transforming growth factor-beta-1
TGF-β1	transforming growth factor-beta-3
HLA	human leucocyte antigen
BCN	bicomponent-network
dHAM	decellularized human amniotic membrane
dHAMMA	decellularized human amniotic membrane methacrylate
MA	methacrylic anhydride
GelMAdHAMMA	methacrylated gelatin decellularized Human Amniotic Membrane methacrylate
GelMA	methacrylated gelatin
DE	dermal equivalent
BM-SCs	bone marrow stromal cells
hDAM	human dehydrated amniotic membrane
MSS	Manchester Scar Scale
BM-SCs-hDAM	bone marrow stromal cells human dehydrated amniotic membrane
AV	aloe vera
AME	amniotic membrane extract
HAAM	human acellular amniotic membrane
dHACA	human amnion and chorion allograft
TESS	skin tissue engineering substitutes
DFU	diabetic foot ulcer
hAMSCs-CM	media conditioned by human amniotic mesenchymal stem cells
hAECs-CM	media conditioned by human amniotic epithelial stem cells
LOXL2	lysyl-oxidase-2
JNK	c-jun N-terminal kinase
WJ-MSC	Wharton’s jelly mesenchymal stem cells
DF	dermal fibroblast
MSCs	mesenchymal Stem Cell
MenSCs	menstrual blood-derived stem cell
DAM	decellularized human amniotic membrane
MenSCs-DAM	menstrual blood-derived stem cell decellularized human amniotic membrane
hAAM	human acellular amniotic membrane
rHFSC	rat hair follicle stem cell
EdU	5-ethynyl-2-deoxyuridine
H&E	hematoxylin and eosin
rHFSChAAM	rat hair follicle stem cells human acellular amniotic membrane
iPSC	induced pluripotent stem cells
EpSCs	epithelial stem cells
iPSC-EpSC	induced pluripotent stem cells- epithelial stem cells
ADMSC	adipose-derived mesenchymal stem cells
AAM	acellular amniotic membrane
STSG	split-thickness skin grafts
HD-AM	hyperdry amniotic membrane
BMSC	bone marrow stem cells
BAWD	bioactive wound dressing
AME	amniotic membrane extract
PMN	polymorphonuclear
TE	tissue engineering
PMNL	human polymorphonuclear cells
TNFα	tumor necrosis factor-alpha
COLI	collagen I
MMP3	matrix metalloproteinase-3
ESF	electrospun nanofiber silk fibroin
AT-MSCs	adipose tissue-derived mesenchymal stem cells
VEGFa	vascular endothelial growth factor
bFGF	basic fibroblast growth factor
RT-PCR	reverse transcriptase polymerase chain reaction
UC-MSC	umbilical cord mesenchymal stem cells
FGF-7	fibroblast growth factor 7
RSF	random skin flaps
BM-MSCs	bone marrow mesenchymal stem cells
JEB	junctional epidermolysis bullosa
EB	epidermolysis bullosa
SEC	skin epithelial cell sheet
cryo-SEC-AM	cryopreserved skin epithelial cell sheet amniotic membrane
VAS	visual analog scale
ASA	amniotic suspension allograft
